# The relationship between pregnancy stress and antepartum depression in Chinese pregnant women: the mediating effect of social support and maternal health literacy

**DOI:** 10.3389/fpsyt.2025.1600448

**Published:** 2025-06-23

**Authors:** Ling Zhang, Fanghui Wu, Fengju Li, Xinyi Feng, Hong Yang, Tao Zhou, Honghui Rong, Chuanfen Zheng, Ting Luo, Lu Lu, Enyu Lei, Guangxu Deng, Li Ren, Ji-An Chen

**Affiliations:** ^1^ Department of Health Education, College of Military Preventive Medicine, Army Medical University, Chongqing, China; ^2^ Department of Basic Psychology, School of Psychology, Army Medical University, Chongqing, China; ^3^ College of Basic Medical Sciences, Army Medical University, Chongqing, China; ^4^ Department of Obstetrics and Gynecology, Chongqing Health Center for Women and Children, Chongqing, China

**Keywords:** depression, stress, social support, health literacy, structural equation modeling

## Abstract

**Objective:**

This study aims to explore the relationship between pregnancy stress (PS) and antenatal depression (AD) among Chinese pregnant women and to investigate whether maternal health literacy (MHL) and social support (SS) mediate the relationship between PS and AD.

**Method:**

This cross-sectional study utilized a two-stage sampling approach, combining stratified random cluster sampling in the first stage with convenience sampling in the second stage, to enroll 1,391 pregnant women attending prenatal care at six hospitals in Chongqing between September 2023 and February 2024. Data were collected using structured scales, including general information questionnaire, Edinburgh Postnatal Depression Scale(EPDS), Pregnancy Stress Scale(PSS), Multidimensional Scale of Perceived Social Support(MSPSS) and Maternal health literacy questionnaire. We utilized Amos 22.0 to establish a structural equation model(SEM).

**Results:**

443 participants (31.85%) reported symptoms of depression, and 382 participants (27.46%) had moderate or above stress; in contrast, only 82 participants (5.90%) reached the level of MHL. Spearman correlation analysis revealed that all the variables were significantly correlated with AD (*p* < 0.01). SEM revealed several effects on AD, including PS (*β* = 0.470, *p* < 0.01), SS (*β* = -0.257, *p* < 0.01) and MHL (*β* = -0.095, *p* < 0.01), all of which were associated with higher depression scores. SS and MHL mediated the link between PS and AD. MHL and SS exerted a negative influence on PS (*β* =−0.236, -0.289; *p* < 0.01). There was no evidence of a mediating effect of MHL on the relationship between SS and AD.

**Conclusion:**

PS, SS and MHL have a direct effect on AD. Moreover, MHL and SS play a mediating role between PS and AD. Health care providers should be aware of the potential threat of PS progressing to AD. Under the guidance of the principles of enhancing MHL and strengthening SS, in the short term, the focus should be on improving the MHL among Chinese population.

## 1 Introduction

Owing to changes in hormone levels and psychosocial factors (1), antepartum depression (AD) is becoming one of the most common complications during pregnancy (2). A comprehensive review of the global prevalence of AD reported that the overall prevalence of AD is 15–65% in low- and middle-income countries (3) and 5–30% in developed countries (4). In China, the prevalence of AD is approximately 10.3%~54.8% (1, 5). This proportion might, in fact, be significantly greater due to the stigma associated with depression, which makes women hesitate to share signs of sadness and irritability (6). Moreover, they may erroneously believe that hormonal protection during pregnancy shields them from psychological disturbances, leading to depression often being overlooked or underestimated (7). AD poses potential risks to both maternal and newborn health. Not only can it lead to miscarriage, premature delivery, low birth weight, and fetal distress (8), it may also predispose infants to later life issues such as temperament difficulties in infancy, emotional and behavioral problems in childhood, and mental health problems during adolescence (9, 10). Moreover, AD has also been recognized as the strongest predictor of postpartum depression in many studies (11–13). In summary, this evidence highlights the importance of focusing on the antenatal stage for the development of preventive and therapeutic interventions (11).

There are two possible mechanisms for explaining maternal depression: biological and psychosocial. From a psychosocial perspective, the strongest predictor is severe life stress (14). Pregnancy, as a potential stressor, is characterized mainly by changes and concerns in various aspects (15) which are collectively referred to as pregnancy stress (PS). PS is a triggering factor that exacerbates depression in pregnant women (16). A previous study revealed that 6.0–16.7% of pregnant women have high levels of perceived stress, whereas 13.6–91.86% of pregnant women have mild–moderate perceived stress (17, 18). The prevalence of PS can reach 94.2% in some regions of China (19), suggesting that a significant majority of pregnant women endure PS during gestation. PS may increase anxiety and disrupt sleep quality, thereby increasing the likelihood of AD (14, 20, 21). These findings underscore the importance of identifying methods to regulate stress and prevent depression (14).

Under the framework of stress process theory, mediating factors such as social support (SS), particular behavioral strategies, and cognitive strategies in coping are key factors (14, 22). These factors can influence individuals’ subjective PS experiences, thereby shaping their subsequent actions and efficiently regulating the impact of stress during pregnancy. This theory suggests that SS significantly shapes how we perceive and handle stress (23). When we feel supported and capable of coping, stress decreases. Research results have indicated a negative correlation between SS and PS (24, 25). Low SS plays a crucial predictive role in severe PS (17). Some studies have also suggested that there is a positive correlation between greater SS and improved AD (26, 27). SS is beneficial for both physical and mental health regardless of whether an individual is under pressure (25). However, whether SS plays a mediating role in the relationship between PS and AD has been less explored.

In addition, improving health literacy (HL) has become an important goal in the field of health in China. HL is generally defined as the ability of an individual to obtain, process, and understand basic health information and services to make appropriate health decisions. HL is an important factor in determining health (28), and this importance has driven an increasing amount of research on HL. Related studies have revealed a relationship between HL and perceived stress levels (29). People with high HL have lower perceptions of stress (30). HL is also related to the incidence rate of depression (31, 32). People with low HL may fail to recognize the symptoms and signs of depression and may not seek professional help (33, 34).

However, there is a lack of research on the correlation between maternal health literacy (MHL) and AD, as well as on the correlation between MHL and SS. There have been no reports on the mediating effect of MHL on the relationship between PS and AD. Given the limited understanding of the synergistic mechanism of AD and its related factors, this study aims to construct a structural equation model (SEM) to analyze the complex relationships among AD, MHL, PS, and SS and, in particular, to explore the impact pathway of PS on AD and the potential mediating roles of MHL and SS, which can provide a theoretical basis and intervention ideas to ameliorate AD.

## 2 Methods

### 2.1 Study design and participants

This cross-sectional survey was conducted from September 2023 to February 2024 among pregnant women in Chongqing, China, using a two-stage sampling approach. In the first stage, we implemented stratified random cluster sampling across three geographical regions (1): Two districts were randomly selected from ten in northeastern Chongqing (2); One district was randomly selected from six in southeastern Chongqing (3); Three districts were selected from twenty-one in the One-Hour Economic Circle region. In the second stage, convenience sampling was used to distribute questionnaires to pregnant women at hospitals in the six selected districts during the survey period, ensuring that each pregnant woman had an equal likelihood of being selected. The inclusion criteria were as follows (1): at least 18 years old (2), permanent residence in Chongqing (3), singleton pregnancy, and (4) no cognitive dysfunction or previous history of mental disorders. During this study, the investigators explained the objectives and importance of the questionnaire survey to all participants, who provided their written informed consent and were reminded of their right to withdraw at any stage of the study. Answers to the questionnaire were collected either through face-to-face interviews or through self-administered questionnaires completed by the literate participants. A total of 1422 pregnant women agreed to participate and returned questionnaires, of which 31 questionnaires were excluded because of invalid or incomplete responses. Ultimately, 1391 valid questionnaires were included in the analysis, representing a survey completion rate of 97.82%.

### 2.2 Instruments

#### 2.2.1 General information questionnaire

A baseline questionnaire was designed to collect pregnant women’s general information, such as age, maternal and paternal education, monthly family income, occupation type, week of gestation, unplanned pregnancy status, sleep status, health status, marital relationship, and whether they had gone to maternity school.

#### 2.2.2 Antepartum depression

AD was measured via the Edinburgh Postnatal Depression Scale (EPDS) developed by Cox et al. (35) and translated into Chinese by Lee et al. (36). It has also been validated for screening depression during pregnancy (37–39). The scale consists of 10 items. Each item is scored with a value from 0–3, with possible total scores ranging from 0–30. Previous studies have established that a cutoff score of 10 on the Chinese version of the EPDS demonstrates clinical utility in identifying probable depression (20, 39). In the present study, the Cronbach’s α of the EPDS was 0.851.

#### 2.2.3 Pregnancy stress scale

The Pregnancy Stress Scale (PSS), a self-report assessment tool devised by Chen et al. in Taiwan (40), has been extensively utilized in relevant research among pregnant Chinese women to evaluate their PS status (1, 8, 19, 20). It consists of 30 items categorized into 4 factors: parenthood recognition (15 items), maternal and child health and safety (8 items), changes in body shape and physical activity (4 items), and other stressors (3 items), each using a 4-point Likert scale (17). The average score of all the items is calculated to assess the level of prenatal stress, and higher scores indicate higher levels. The Cronbach’s α internal consistency rating of the PS in this study was 0.94.

#### 2.2.4 Multidimensional scale of perceived social support

The Multidimensional Scale of Perceived Social Support (MSPSS) is a 12-item instrument for measuring perceived SS that was developed by Zimet et al. (41). The Chinese version of the MSPSS has shown good reliability and validity in the literature (42, 43). Participants rated their responses on a seven-point Likert response scale (from 1 = very strongly disagree to 7 = very strongly agree). The scale was divided into 3 parts: family, friends, and significant others (43). The possible scores range from 4–28 for each subscale and from 12–84 for all items, with higher scores indicating greater total perceived social support. The MSPSS had a Cronbach’s α of 0.92 in the current study.

#### 2.2.5 Maternal health literacy questionnaire

The MHL questionnaire, developed and compiled by the China Maternal and Child Health Care Association, is based on Article 55 of Basic Knowledge and Skills of Maternal and Child Health Literacy (44, 45). This questionnaire, which is designed to assess crucial health aspects, encompasses 35 inquiries into nutrition and physical activity during pregnancy, weight management strategies, childbirth modalities, breastfeeding practices, neonatal care techniques, and numerous other vital health concerns. It is categorized into three key domains: basic knowledge of mother and infant (BKMI), consisting of 22 items; behaviors and lifestyle (BAL), with 8 items; and health-related skills (HRS), encompassing 5 items. A score of 0 denoted an incorrect answer, with the overall HL score ranging from 0–100. Participants who achieved 80% or more of the total score (≥80) were considered to have adequate HL. This threshold was established based on scientific consensus and practical considerations (1): It has been widely adopted in prior research, effectively distinguishing between individuals with strong health skills from those with limited skills (46, 47) (2); This standard was developed by adapting international practices (where 75% is commonly used for scientific literacy assessments) to China’s specific context. The slightly higher threshold reflects both the increased emphasis on fundamental health knowledge and alignment with national policy priorities, particularly the health literacy improvement goals outlined in China’s Healthy China Initiative.

### 2.3 Statistical analyses

EpiData 3.02 was used for double entry of questionnaires, and data processing and analysis were performed with SPSS 26.0. The general characteristics of the participants were assessed using descriptive statistics. Continuous variables are presented as the means and standard deviations, and categorical variables are presented as frequencies/percentages. Comparisons were tested using t tests, analysis of variance (ANOVA), or nonparametric tests. An IBM SPSS Amos22.0 modeling and analysis system was used to establish a path analysis diagram of the relationships among the factors. The following indices were used to evaluate the goodness of fit of the model: the comparative fit index (CFI) >0.9, adjusted goodness-of-fit index (AGFI) >0.9, goodness-of-fit index (GFI)>0.9, normed fit index (NFI) >0.9, incremental fit index (IFI) >0.9, and root mean square error of approximation (RMSEA) <0.08. P < 0.05 was considered statistically significant.

## 3 Results

### 3.1 Baseline characteristics

Among the 1422 questionnaires collected, 31 had invalid or incomplete responses and were excluded from the study. Therefore, a total of 1391 pregnant women aged between 18~44 (29.63 ± 4.15) were included in the study. The sociodemographic characteristics of the study participants are displayed in **Table 1**. Among the participants, 1196 (85.98%) were between 18–35 years old, and 195 (14.02%) were older than 35 years old. A total of 1213 (87.20%) lived in central urban districts, and 195 (14.02%) lived in urban areas. Most of them were Han (87.20%). In total, 1020 (73.33%) were employed, and 371 (26.67%) were housewives. The proportions of primigravidas and multiparous women were 55.72% and 44.28%, respectively. Most of them had not attended maternity school (61.68%). More than half of the participants had good sleep (67.29%), and most of the participants were in good health (72.32%).

**Table 1:** MHL, AD, SS and PS scores of pregnant women with different demographic characteristics (n = 1391).

Characteristics	Categories	N(%)	MHL	AD	SS	PS
Age	<35	1196 (85.98)	43.36 ± 19.63	7.57 ± 4.32	62.92 ± 13.10	0.76 ± 0.49
	≥35	195 (14.02)	45.95 ± 18.19	7.74 ± 4.80	61.63 ± 14.71	0.71 ± 0.51
	*p value*		0.085	0.618	0.210	0.205
Residence	town groups	445 (31.99)	46.70 ± 22.86	7.28 ± 4.44	59.70 ± 14.34	0.69 ± 0.55
	central urban district	956 (68.01)	42.33 ± 17.46	7.75 ± 4.36	64.17 ± 12.60	0.79 ± 0.46
	*p value*		0.000	0.066	0.000	0.001
Ethnicity	Han	1213 (87.20)	44.08 ± 19.84	7.53 ± 4.44	62.76 ± 13.36	0.75 ± 0.49
	Others	178 (12.80)	41.30 ± 16.39	8.08 ± 4.05	62.62 ± 13.24	0.79 ± 0.51
	*p value*		0.041	0.114	0.894	0.320
Marital status	Unmarried/Divorce	37 (2.66)	42.08 ± 22.74	7.78 ± 4.56	57.84 ± 14.86	0.72 ± 0.56
	married	1354 (97.34)	43.77 ± 19.36	7.59 ± 4.39	62.88 ± 13.28	0.76 ± 0.49
	*p value*		0.602	0.794	0.023	0.608
Participants’ education	Below primary school	27 (1.94)	35.26 ± 17.58	7.37 ± 4.45	56.89 ± 12.43	0.69 ± 0.34
	Primary school	156 (11.21)	37.83 ± 19.38	8.28 ± 4.61	57.40 ± 13.68	0.72 ± 0.48
	High school	279 (20.06)	43.04 ± 20.60	7.64 ± 4.73	57.81 ± 14.68	0.75 ± 0.50
	University or higher	929 (66.79)	45.17 ± 18.92	7.48 ± 4.24	65.29 ± 12.12	0.77 ± 0.49
	*p value*		0.000	0.206	0.000	0.566
Participants’ occupation	Unemployed/housewives	371 (26.67)	40.96 ± 19.46	7.92 ± 4.23	59.06 ± 14.06	0.72 ± 0.48
	Employed	1020 (73.33)	44.73 ± 19.36	7.48 ± 4.45	64.08 ± 12.81	0.77 ± 0.50
	*p value*		0.001	0.100	0.000	0.109
Monthly income (CNY)	<5000 RMB	263 (18.91)	41.94 ± 20.58	8.09 ± 4.64	57.28 ± 14.93	0.73 ± 0.51
	5000~7999RMB	460 (33.07)	44.24 ± 20.61	7.21 ± 4.22	62.58 ± 12.91	0.77 ± 0.50
	8000~14999RMB	453 (32.57)	44.04 ± 17.98	7.71 ± 4.54	64.15 ± 12.77	0.78 ± 0.49
	≥15000RMB	215 (15.46)	44.16 ± 18.44	7.60 ± 4.07	66.81 ± 11.12	0.73 ± 0.44
	*p value*		0.432	0.069	0.000	0.451
Health insurance	No	345 (24.80)	39.01 ± 19.69	8.30 ± 4.44	60.14 ± 14.26	0.71 ± 0.47
	Yes	1046 (75.20)	45.28 ± 19.13	7.37 ± 4.35	63.60 ± 12.92	0.77 ± 0.50
	*p value*		0.000	0.001	0.000	0.050
Gestational weeks	The first trimester	335 (24.08)	41.69 ± 22.27	7.17 ± 4.31	63.33 ± 13.19	0.70 ± 0.52
	The second trimester	376 (27.03)	40.62 ± 18.32	7.72 ± 3.97	64.81 ± 12.34	0.77 ± 0.48
	The third trimester	680 (48.89)	46.44 ± 18.18	7.74 ± 4.63	61.31 ± 13.79	0.78 ± 0.48
	*p value*		0.000	0.119	0.000	0.078
Abnormal pregnancy history	No	1056 (75.92)	43.88 ± 19.79	7.41 ± 4.41	62.54 ± 13.58	0.74 ± 0.49
	Yes	335 (24.08)	43.25 ± 18.38	8.18 ± 4.27	63.38 ± 12.56	0.80 ± 0.50
	*p value*		0.608	0.005	0.316	0.056
Parity	Primigravida	775 (55.72)	42.27 ± 19.15	7.52 ± 4.18	64.11 ± 12.56	0.77 ± 0.51
	Multipara	616 (44.28)	45.56 ± 19.69	7.69 ± 4.64	61.03 ± 14.08	0.75 ± 0.47
	*p value*		0.002	0.477	0.000	0.464
Participated in pregnancy school	Never	858 (61.68)	40.39 ± 18.70	7.63 ± 4.35	63.44 ± 13.11	0.75 ± 0.5
	1~2 times	393 (28.25)	46.81 ± 19.15	7.83 ± 4.38	61.37 ± 12.94	0.79 ± 0.48
	≥3 times	140 (10.06)	55.49 ± 18.91	6.72 ± 4.61	62.34 ± 15.5	0.70 ± 0.51
	*p value*		0.000	0.034	0.037	0.169
Sleeping status	Terrible	455 (32.71)	42.31 ± 17.37	9.10 ± 4.68	61.42 ± 14.36	0.78 ± 0.50
	Good	936 (67.29)	44.42 ± 20.36	6.87 ± 4.05	63.39 ± 12.78	0.75 ± 0.49
	*p value*		0.058	0.000	0.013	0.220
Self-evaluated health status	Good	1006 (72.32)	44.95 ± 20.14	6.92 ± 4.26	63.00 ± 13.45	0.74 ± 0.50
	Middle	369 (26.53)	40.38 ± 16.90	9.33 ± 4.17	62.10 ± 12.99	0.80 ± 0.46
	Terrible	16 (1.15)	44.13 ± 22.17	10.5 ± 5.61	61.38 ± 14.75	0.77 ± 0.34
	*p value*		0.001	0.000	0.497	0.201

### 3.2 Maternal health literacy, antepartum depression, social support, pregnancy stress level and population distribution of scores

In this study, 82 participants (5.90%) reached the level of MHL, and 443 participants (31.85%) exhibited symptoms of AD. A total of 1287 participants (92.52%) reported stress, of which 382 (27.46%) had moderate or above stress. There were statistically significant differences in the MHL score among pregnant women, with differences in places of residence, nationality, educational level, occupation, health insurance, different gestational weeks, primigravida status, participation in maternity schools and self-evaluated health status(*p < 0.05*). There were statistically significant differences in AD among pregnant women with insurance, abnormal pregnancy history, participation in maternity schools, sleeping status and self-evaluated health status*(p < 0.05)*. In addition to age, ethnicity, abnormal pregnancy history and self-evaluated health status, SS was significantly different across different demographics*(p < 0.05)*. In contrast, PS was significantly different only in residential areas*(p < 0.05)*, as shown in **Table 1**.

### 3.3 Description of MHL, SS, and PS with and without depression symptoms

In this study, the AD score was (7.60 ± 4.39). The MHL score of pregnant women was (43.73 ± 19.45), with scores for BKMI, BAL, and HRS being (30.54 ± 13.55; 7.88 ± 4.98; 5.30 ± 3.13). The SS score was (62.74 ± 13.34), with family support, friend support, and other support scores being (21.60 ± 4.76; 20.72 ± 4.73; 20.42 ± 4.72). The PS score was (0.76 ± 0.79), with scores for PR, MCHS, BSPA and OS being (0.46 ± 0.42; 1.01 ± 0.63; 0.87 ± 0.68; 0.68 ± 0.59). Among all the variables, the difference in scores between depressed and non-depressed patients was statistically significant(*p < 0.05*), as shown in **Table 2**.

**Table 2:** Description of MHL, SS, and PS with and without depression symptoms (n = 1391).

Variables	Mean ± SD	Depression	Nondepression	Z Value	p Value
AD	7.60 ± 4.39	12.44 ± 2.83	5.33 ± 2.91	-31.971	0.000
MHL	43.73 ± 19.45	40.34 ± 17.14	45.31 ± 20.26	-3.895	0.000
BKMI	30.54 ± 13.55	28.48 ± 12.75	31.51 ± 13.80	-3.487	0.000
BAL	7.88 ± 4.98	6.92 ± 4.06	8.32 ± 5.29	-4.120	0.000
HRS	5.30 ± 3.13	4.93 ± 2.86	5.48 ± 3.23	-2.513	0.012
SS	62.74 ± 13.34	56.75 ± 13.36	65.54 ± 12.38	-30.173	0.000
Family	21.60 ± 4.76	19.62 ± 5.00	22.53 ± 4.35	-11.976	0.000
Friends	20.72 ± 4.73	18.66 ± 4.82	21.68 ± 4.37	-10.848	0.000
Others	20.42 ± 4.73	18.48 ± 4.65	21.33 ± 4.49	-11.244	0.000
PS	0.76 ± 0.49	0.99 ± 0.50	0.65 ± 0.45	-10.980	0.000
PR	0.46 ± 0.42	0.66 ± 0.46	0.37 ± 0.37	-12.596	0.000
MCHS	1.01 ± 0.63	1.27 ± 0.65	0.88 ± 0.58	-12.477	0.000
BSPA	0.87 ± 0.68	1.11 ± 0.71	0.76 ± 0.64	-10.599	0.000
OS	0.68 ± 0.59	0.91 ± 0.63	0.57 ± 0.53	-9.436	0.000

### 3.4 Correlations among MHL, SS, PS and AD

**Table 3** shows the results of the spearman correlation analyses of MHL, SS, PS and AD. AD was negatively correlated with MHL and SS (*p < 0.05*). MHL was negatively correlated with PS (*p < 0.05*) but not with SS (*p > 0.05*). There was a positive correlation between PS and AD (*p < 0.05*).

**Table 3:** Correlations (r) among MHL, SS, PS and AD.

Variables	AD	MHL	SS	PS
AD	1			
MHL	-0.126^**^	1		
SS	-0.364^**^	0.003	1	
PS	0.418^**^	-0.126^**^	-0.296^**^	1

^**^P < 0.01 (double tails), the correlation was signiﬁcant.

### 3.5 Pathway analysis of AD in pregnant women

**Figure 1:**
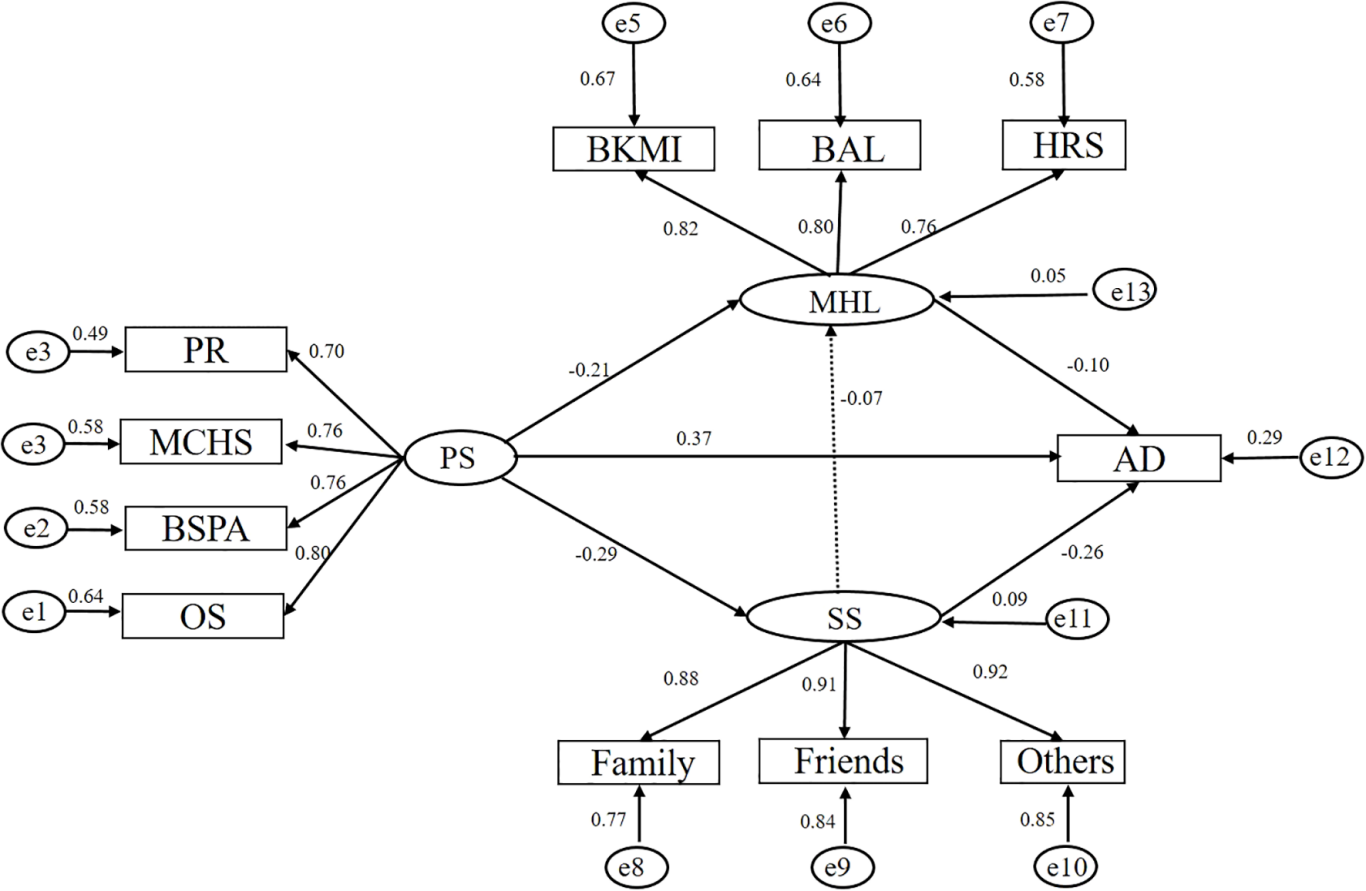
Standardized estimates of relationships and effect sizes in the structural model.

On the basis of the relevant literature and professional knowledge, a preliminary theoretical model was constructed by using the Bootstrap mediating effect test. After the analysis of the mediating effect, it was found that there was no statistically significant difference between MHL and SS(*p > 0.05*). Therefore, to better fit the structural model, we eliminated the direct path. **Figure 1** shows the final model with standardized path coefficients that have sufficient fit. The overall fit of this model was as follows: CFI = 0.991, GFI = 0.986, NFI = 0.986, IFI = 0.991, AGFI = 0.976, RMSEA = 0.036. There were significant correlations between the observed variables and their respective latent variables, with the majority of regression weights exceeding 0.7, suggesting a strong linkage.

**Table 4** presents the standardized coefficients of the path analysis. The results of the path analysis revealed that PS had a positive influence on AD (*β* = 0.375, *p* < 0.05) and a negative influence on MHL and SS (β =−0.236, -0.289; p < 0.05). SS had a negative effect on AD (*β* = -0.257, *p* < 0.05) but had no statistically significant effect on MHL (*p* > 0.05). MHL had a negative influence on AD (*β* =−0.095, *p* < 0.05). According to the analysis conducted with the latent variables, it revealed two significant mediating pathways (1): The PS→MHL→AD pathway showed an indirect effect of 0.020 (*β* = -0.213 × -0.095), accounting for 4.3% of the total effect (2); The PS→SS→AD pathway demonstrated an indirect effect of 0.074 (*β* = 0.287 × 0.257), representing 15.8% of the total effect (0.468). These findings indicate that both MHL and SS serve as partial mediators in the relationship between PS and AD, with SS exhibiting a relatively stronger mediating effect than MHL.

**Table 4:** Standardized direct, indirect, and total effects of all study pathways (n = 1391).

Relationship between variables	Direct Effects	Indirect Effects	Total Effects
H1: PS→MHL	-0.213	0.000	-0.213
H2: PS→SS	-0.287	0.014	-0.287
H3:PS→AD	0.374	0.095	0.468
H5:MHL→AD	-0.095	0.000	-0.095
H6:SS→AD	-0.256	0.007	-0.256

## 4 Discussion

The prevalence of AD in this study was 31.85%, which was slightly higher than that reported in a multisite survey in China (28.4%) (48) and lower than that reported in Nigeria (45.2%) (49) and Afghanistan (42.8%) (50). Experiencing major events can have significant psychological impacts (50). During a survey conducted in Urumqi, Xinjiang, China, during the COVID-19 pandemic, researchers reported that the prevalence rate of AD was 58.9% (51). Therefore, when dealing with major public health emergencies, it is imperative not only to properly execute various response efforts but also to pay attention to addressing the impact on the population’s mental health as the timely detection of and effective intervention in AD are crucial.

According to the absolute value of the path coefficient, the most important factor affecting AD was PS, which is consistent with relevant reports. Some studies in China have confirmed that the psychological stress experienced by pregnant women is a major factor in prenatal depression (14, 51). In our study, the prevalence of stress was 92.52%, of which 27.46% of participants had moderate or greater stress. The reason behind the higher PS among our study participants, compared with many similar studies conducted elsewhere, may be attributed to their heightened concerns about the impact of the COVID-19 pandemic on their own health and the well-being of their babies, especially following the comprehensive lifting of restrictions related to the pandemic in our country. Among the several dimensions of stress in our study, the scores for maternal and child health and safety were the highest. This finding also suggests that when confronted with the stressful experience of pregnancy, women inevitably harbor concerns for both their own well-being and that of the fetus, and these concerns serve as the main factors influencing pregnancy-related stress, which is consistent with the results of previous studies in China and Japan (51, 52). Hence, alleviating the psychological stress of women during pregnancy is an important factor in preventing AD.

In this research, there was a significant negative correlation between PS and SS. Previous study in China had indicated that SS not only effectively alleviates the negative emotions of pregnant women caused by adverse events but also strengthens their personal coping capabilities (11). SS plays an important mediating role between PS and AD. PS has an indirect effect on AD through SS, that is, PS may reduce an individual’s perceived SS, which in turn increases the risk of anxiety and AD. In the study, PS is negatively correlated with SS, while a lower SS is positively correlated with an increased severity of AD, indicating that PS can also indirectly affect the risk of AD. These results are consistent with existing evidence in China that inadequate social support, including lack of emotional support, or practical assistance, serves as a critical pathway linking PS to AD development (53). Moreover, the level of SS serves as a protective factor against AD; that is, the more SS that pregnant women perceive, the less severe their AD will be. This finding is inconsistent with the results reported in a previous study in China (20), but similar to those found in other studies in China as well (11). In this study, those pregnant women who did not report AD had higher scores than those of the depressed group in all three dimensions of SS. Furthermore, although family members remain the primary source of SS for pregnant women, support from friends and colleagues should not be overlooked. Relevant studies have shown that family support significantly influences the mental health of pregnant women, whereas friends and colleagues can offer additional emotional and informational support (20). Therefore, medical staff can commence from the perspective of social support; focus on the demographic characteristics that affect SS; and make full use of SS from family members, peers, medical staff, etc. These staff should provide high-risk pregnant women with information, materials, companionship, and emotional support; adjust their perception of stressors; help relieve PS; and prevent the occurrence of AD.

The results of this study indicated a negative correlation between MHL and PS. In other words, the higher the MHL is, the weaker the perception of stress. Similar findings have been reported in Greece and Iran (29, 30). Pregnant women with higher MHL tend to better understand and cope with various physiological and psychological changes during pregnancy, which enables them to effectively reduce PS. In contrast, MHL was significantly negatively associated with AD, which is inconsistent with the findings of individual study in Iran (30); nevertheless, most studies from the United States, China and Taiwan still support the idea that there is a negative correlation between HL and depression (8, 54, 55). This correlation may be because individuals with higher HL levels are able to recognize their own emotional problems or stress and actively access health information or receive help from health care providers. Additionally, path analysis revealed that MHL plays an intermediary role in PS and AD. In other words, people with HL can regulate stress in a timely manner and thus reduce the risk of depression. Therefore, health care workers during pregnancy should pay attention to improving the HL of pregnant women. Strengthening health education facilitates the provision of scientific pregnancy knowledge and psychological adjustment skills to help pregnant women establish appropriate health concepts and improve their ability to manage themselves and cope with stress.

In our research, there was no significant correlation between MHL and SS. This result is inconsistent with previous studies in China (56, 57). Prior literature suggests two possible explanations for why a correlation might be expected: first, when MHL is low, SS may compensate by filling knowledge gaps. Second, mothers with high MHL may be more adept at recognizing and utilizing SS resources, making them more likely to seek help when needed. While these mechanisms might theoretically explain a potential correlation, our findings showed no such correlation. This lack of correlation may be due to differences in the HL assessment scale. The scale used in this study was specifically designed for maternal and infant HL, which reflects the nutritional, safety, first aid and medical knowledge of pregnant women, all of which were obtained from health care professionals. However, most family members do not have a medical background in China (58). It is worth noting that in certain cultural contexts (such as Asian), the influence of social support on pregnant women may be more reflected in emotional support and practical care (e.g., specific behaviors like dietary adjustment and sharing of household chores), rather than in health information acquisition or participation in medical decision-making. These differences in support mechanisms may result in a more direct impact on behavioral compliance and psychological adaptability rather than directly enhancing health literacy. To some extent, this explains the possible reasons for the lack of a significant correlation between social support and the level of health literacy in this study. Although this study found no direct association between social support and health literacy, family support remains an important part of prenatal care. Future studies can further distinguish the types of support (such as emotional support vs. information support) and their impacts on health literacy in different dimensions. In addition, the overall level of MHL in this survey was relatively low. Follow-up studies can be grouped according to the level of MHL to further analyze the correlations among them. In light of these considerations, we plan to continue to explore the relationship between MHL and SS in future studies to reveal the intrinsic relationship more comprehensively.

## 5 Strengths and limitations

This study focused on multiple variables related to AD in pregnant women, including MHL, SS, and PS. Its main contribution is its groundbreaking establishment of the relationships among these variables, providing a theoretical basis and intervention directions for improving the AD situation of pregnant women. Moreover, the study adopted a standardized questionnaire to comprehensively evaluate MHL, SS, AD, and PS. This approach has created a replicable framework for future research in similar fields. However, this study also has certain limitations. First, owing to the adoption of a cross-sectional research method, this makes it difficult for us to determine the causal relationship among PS, MHL, SS and AD. It is particularly necessary to point out that when examining the mediating variables, since all the variables are measured at the same time point, we cannot clearly define the temporal sequence and dynamic change process among the variables. Future research can conduct longitudinal studies, measuring these variables multiple times at different stages of pregnancy to verify the mediating mechanism more accurately. Second, owing to the numerous questions in the questionnaire, it took a long time to obtain results. In the future, shorter versions of the questionnaires can be used. Additionally, this study relied only on one administration of the EPDS scale to assess AD and had no participation from psychiatrists in the diagnosis; since only presenting depressive symptoms were assessed, this approach may have overlooked some women with depressive symptoms, resulting in false negatives. In future research, secondary verification can be conducted in combination with clinical interviews.

## 6 Conclusions

In this study, we explored several factors related to AD in pregnant women. It was determined that PS, SS, and MHL directly influence AD, with PS having the most important impact and thus emerging as a pivotal factor in AD. Our research also revealed the regulatory functions of MHL and SS between PS and AD, further enriching our understanding of the intricate mechanisms underlying the mental health of pregnant women. On the basis of these findings, we provide targeted recommendations for health care providers. First, health care workers should be aware of the potential threat posed by the progression of PS to AD among pregnant women. Second, it is proposed to adopt the enhancement of MHL and the reinforcement of SS as guiding principles. Considering that SS is difficult to change in a short period, this study suggests that professional medical staff should pay attention to improving the MHL of pregnant women.

## Data Availability

The raw data supporting the conclusions of this article will be made available by the authors, without undue reservation.
